# Factors associated with household food insecurity and dietary diversity among day laborers amid the COVID-19 pandemic in Bangladesh

**DOI:** 10.1186/s40795-022-00517-8

**Published:** 2022-03-23

**Authors:** Md. Hasan Al Banna, Abu Sayeed, Satyajit Kundu, Anna Kagstrom, Mst. Sadia Sultana, Musammet Rasheda Begum, Md Shafiqul Islam Khan

**Affiliations:** 1 Department of Food Microbiology, Faculty of Nutrition and Food Science, Patuakhali Science and Technology University, Patuakhali, 8602 Bangladesh; 2 Department of Post-Harvest Technology and Marketing, Patuakhali Science and Technology University, Patuakhali, 8602 Bangladesh; 3 School of Public Health, Southeast University, Nanjing, 210009 China; 4 Faculty of Nutrition and Food Science, Patuakhali Science and Technology University, Patuakhali, 8602 Bangladesh; 5 Department of Public Mental Health, National Institute of Mental Health, Topolová 748, Klecany, 250 67 Czech Republic; 6 Department of Psychiatry and Medical Psychology, Third Faculty of Medicine, Charles University, Prague, Czech Republic; 7 Department of Public Health and Informatics, Jahangirnagar University, Savar, Dhaka, Bangladesh; 8 Department of Agricultural Economics and Social Sciences, Chattogram Veterinary and Animal Sciences University, Chattogram, 4225 Bangladesh

**Keywords:** Food insecurity, Dietary diversity, Day laborers, COVID-19, Bangladesh

## Abstract

**Backgrounds:**

Food insecurity and dietary diversity remain a public health concern in developing countries like Bangladesh which is exacerbated by the COVID-19 especially for day laborers’ families in Bangladesh. This study aimed to determine factors associated with household food insecurity and household dietary diversity among day laborers during the COVID-19 pandemic in Bangladesh.

**Methods:**

This cross-sectional study was conducted among 343 households of day laborers in Bangladesh using a semi-structured questionnaire. Household food security (HFS) and Household dietary diversity (HDD) scores were assessed using the HFS scale and household’s 24-h recall of intake of 12 food groups, respectively.

**Results:**

The overall mean scores of HFS and HDD were 26.80 (SD, 4.83) and 4.08 (SD, 1.15). Having household head aged > 40 years and monthly household income > 5000 Bangladeshi Taka (BDT) were positively associated with HDD scores. Having an education level above secondary, monthly household income > 5000 BDT, and having a refrigerator were associated with the higher HFS scores, whereas having family members > 5 was a potential determinant of lower HFS scores. Pandemic-induced work loss and food scarcity were also potential determinants of lower HFS and HDD scores. Approximately 94% of respondents reported their wages were reduced, and 76% were deprived of the same quantity of food as pre-pandemic periods.

**Conclusions:**

Lower socio-economic status and pandemic-induced work loss affect the HFS and HDD. Interventions with financial aid and complemented food distributions, particularly among the wage looser, may improve the HFS and HDD of day laborers.

**Supplementary Information:**

The online version contains supplementary material available at 10.1186/s40795-022-00517-8.

## Background

Globally, around 820 million people face hunger daily, and more than two billion people lack essential micronutrients, threatening their health and life expectancy [1]. The United Nations Decade of Action on Nutrition 2016–2025 and the World Health Organization Sustainable Development Goals (SDGs) for 2030 recognize nutrition and food security as a human right and public health priority and continue the call for urgent global and regional progress in eliminating malnutrition and hunger [2]. The outbreak and course of the COVID-19 pandemic have imposed a new set of challenges, unprecedently affecting the world across all aspects of human life, including public health and well-being [3]. The pandemic has steadily turned from a mere health emergency into an economic crisis, especially in low-and-middle-income countries, posing severe threats to food security and nutrition of global populations in the forms of stay-at-home orders, economic decline, and food system disruptions, including marketing, logistics and trading systems [4]. Thus, the economic turmoil caused by the pandemic has jeopardized physical and financial access to adequate nutrition [5].

Moreover, mobility restrictions implemented by countries to stem the spread of COVID-19 have disrupted economies and food transportation, exacerbating vulnerabilities of populations affected by poverty and malnutrition even prior to the pandemic [6]. A recent report projected that the world is not on track to achieve Zero Hunger and meet global nutrition targets by 2030 [7]. As a result of the health and socio-economic impacts of COVID-19, the food security and nutritional status of high-risk sub-populations, namely individuals living at or below respective poverty lines, are likely to worsen [7]. In response, the UN’s Food and Agriculture Organization urges nations to meet the immediate needs of vulnerable populations, and the Global Humanitarian Response Plan outlines necessary steps in light of the pandemic’s effect on food and agriculture [8].

The World Food Summit in 1996 determined that household food security (HFS) exists when all people, at all times, have physical and economic access to sufficient, safe, and nutritious food that meets their dietary needs and food preferences for an active and healthy life [9]. The Covid-19 pandemic affects the core of food security (e.g., food availability, accessibility, utilization, and stability) by disrupting food systems, threatening household incomes, job security, and limiting physical access to food [10]. Several socio-economic factors associated with household food security and dietary diversity during the COVID-19 pandemic included personal savings, household income, rural resident, occupation status of head of household, and nutrition knowledge of the head of household [11, 12]. More robust information is needed to mitigate the adverse impact of COVID-19 on how vulnerable population groups’ HFS and HDD are affected amid the COVID-19 pandemic.

Considering the adverse impact of the COVID-19 pandemic, several studies have been conducted on HFS and HDD [11, 13–16]. A recent study reported that the proportion of food insecure participants increased by 38% and 44% in Kenya and Uganda, respectively. In contrast, the regular consumption of fruits decreased by about 30% in both countries during the COVID-19 pandemic [14]. A recent Bangladeshi study noted that 90% of households from both urban and rural areas suffered from moderate to severe food insecurity during lockdown [13]. Another Bangladeshi study reported that families’ food insecurity increased by 51.7% during the COVID-19 lockdown period [17]. While several studies in Bangladesh examined the impact of the COVID-19 pandemic on HFS and HDD both from rural and urban households, there is a lack of information on how the pandemic affects low-income groups such as day-laborers.

Recent decades have brought about self-sufficiency in food production, household food security, and sharp economic growth in Bangladesh; however, the COVID-19 pandemic has threatened household head's incomes. The COVID-19 pandemic has adversely impacted on the total food consumption status and the livelihood of the marginal population in Bangladesh [18, 19]. This pandemic has created job insecurity (i.e., lost job and income) mostly for peoples working in the informal sectors in Bangladesh [18]. Particularly vulnerable are those dependent on daily wages that are destabilized due to COVID-19, subsequently affecting their families’ food and nutrition. The food insecurity and lower dietary diversity has increased mostly among low-and-middle income groups in Bangladesh during this pandemic due to the unemployment, lower income and greater poverty [11, 18, 19].

A nationwide survey in Bangladesh revealed that 93% of participants had experienced a loss of earnings, with 54% reporting no income in March 2020 [20]. Thus, individuals who depend on labor income would experience a severe income shock threatening household food security and dietary diversity. A rapid assessment can serve to understand the critical needs of this understudied vulnerable group and subsequently inform the prioritization and development of programs to meet the requirements regarding food security and dietary diversity. Thus, this study was undertaken to explore the household food security and dietary diversity, and associated factors among day laborers during the COVID-19 pandemic in Bangladesh.

## Methods

### Setting, study design, and participants

We conducted a cross-sectional survey of day laborers in both urban and rural areas of Bangladesh from September 7 to October 15, 2020. Food security and dietary diversity of day laborers’ households were assessed via face-to-face interviews. Nine rural and nine urban areas were selected from nine divisions based on convenience of access. Purposive sampling technique was employed for this study.. From 400 participants invited to participate in our survey voluntarily, 360 participants responded, and the response rate was 90%. We excluded incomplete questionnaires in our final analysis, resulting in a total sample size of (*n* =) 343. Inclusion criteria for our study required participants to be Bangladeshi, adult (age ≥ 18 years), head of the household, and occupation as a day laborer (porters, masons, construction workers, transporters, etc.). A day laborer was defined as an unskilled laborer who was hired or paid, or worked on a daily basis [21, 22].

### Data collection procedures

Data were collected by 18 researcher staff who were trained by a field supervisor. We used a semi-structured questionnaire containing a total of 37 close-ended items, which were divided into four sections: demographics, effects of COVID-19 on daily life, household food security, and household dietary diversity. The questionnaire was first developed in English, then translated from English to Bengali by two bilingual researchers. Next, to ensure accuracy and eliminate bias, an independent bilingual expert back-translated and finalized the questionnaire. Finally, the questionnaire was pilot-tested with a small group (36 participants) to ensure its transparency and avoid any useless/repeated questionnaire. During the face-to-face interview, appropriate personal protective equipment was used, and social distance was maintained, while each interview took around 20 to 25 min to complete. Participants were informed about the study background, intent, procedures, confidentiality agreement, and informed consent was obtained prior to the interviews. Signatures were obtained for informed consent, and in cases of illiteracy, verbal informed consent was obtained from participants. Obtaining verbal informed consent from the illiterate participants and the overall study protocol was approved by the Research Ethical Committee (REC) of the Department of Food Microbiology, Patuakhali Science and Technology University, Bangladesh (Approval No: FMB:29/05/2020:07).

### Outcome measures

#### Household food security

The Household Food Security Scale (HHFS) was used to assess the HFS status [23]. This scale measures the household food security of families over the previous month, which was also used in an earlier study in the Bangladeshi context [11]. The HHFS comprises 11 items covering the following topics: i) rice and perishable food, ii) cooking frequency, iii) consumption of snacks and iv) management strategies. A score of 1–5 was assigned to each item response, with higher scores indicating higher food security and a lower score indicating more inadequate food security [23]. The internal consistency of the HHFS scale was adequate (Cronbach's alpha = 0.83).

#### Household dietary diversity

**Fig. 1:**
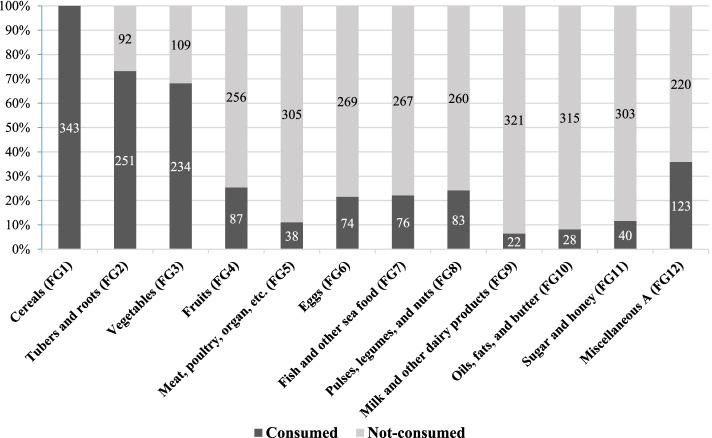
Food groups consumed by Bangladeshi households of day labor’s during the COVID-19 pandemic in the last 24 h (*N* = 343)

The tool for measuring HDD score was adapted from the guidelines of the FANTA project [24]. Another key indicator of HFS is household dietary diversity (HDD), defined as ‘the number of unique foods consumed by household members over a given period’ [24]. HDD is an essential nutrition outcome measuring the variability of household foods during a determined period, reflecting the quality of the household diet [25]. HDD score was assessed by summing up the number of food or food groups consumed by any household member in the 24 h preceding the survey day [11]. Participants were asked about the consumption status (consumed or not consumed) of a total of 12 food groups (see Fig. 1). A score of "1" represents that respondents’ families had consumed food from the respective food group, and "0" was assigned to those who had not consumed the food group in the last 24 h. A sum composite HDD score was created for each household, ranging from 0 to 12, with higher scores suggesting higher dietary diversity. The internal consistency of the HDD scale was sufficient (Cronbach's alpha = 0.79).

### Explanatory variables

The included socio-demographic variables are age (in years), gender, education level, family monthly income (in Bangladeshi *Taka*), place of residence, family size and family type, owning a refrigerator (yes vs. no), and source of nutrition information. Variables related to effects of COVID-19 (self-reported information) on income and occupation of the household head or earning person of the family, food quality, and food quantity consumed by the households were also included in the survey.

### Data analysis

Descriptive statistics were estimated for socio-demographic characteristics of the participants and variables assessing the impact of COVID-19 on respondents’ daily life. A one-way analysis of variance (ANOVA) and independent sample t-tests were employed to distribute HDD and HFS scores and evaluate their mean differences across participants’ characteristics. A multiple linear regression model was used to outline the factors associated with HDD and HFS. The estimates of the strengths of associations were demonstrated by the adjusted beta co-efficient (β) with a 95% confidence interval (95% CI). Multicollinearity was also checked using variance inflation factor (VIF). A p-value of < 0.05 was considered statistically significant. Statistical analyses were performed using the statistical software package SPSS (version 23.0) and STATA (version 17.0).

## Results

### Socio-demographic characteristics of participants

About half of the household heads were from urban areas (49.0%) and above 40 years old (52.5%). Most household heads were male (97.1%), and 21.6% of household heads had no schooling or formal education. Most households were nuclear (88.9%), and 36.2% of households had > 5 family members. About half of the households’ monthly income was below or equal to 5000 BDT (approximately 60 USD), and most households had no refrigerator. About 45.5% of household heads provided no dietary/nutrition information (Table 1).

**Table 1:** Distribution of household dietary diversity and household food security scores across socio-demographic characteristics of participants (*N* = 343)

Variables	N (%)	HDD score			HFS score		
		**M**	**SD**	**p**	**M**	**SD**	**p**
**Residence**							
Rural	175 (51.0)	4.09	1.13	0.908	26.27	4.68	**0.036**
Urban	168 (49.0)	4.07	1.16		27.36	4.92	
**Age in years**							
≤ 40 years	163 (47.5)	3.93	1.08	**0.024**	27.28	4.66	0.085
> 40 years	180 (52.5)	4.21	1.20		26.38	4.95	
**Sex of family head**							
Male	333 (97.1)	4.09	1.15	0.436	26.92	4.77	**0.011**
Female	10 (2.90)	3.80	0.79		23.00	5.29	
**Education of family head**							
No schooling	74 (21.6)	4.11	0.90	0.877	26.57	4.02	0.082
Below secondary	261 (76.1)	4.07	1.20		27.00	4.96	
Above secondary	8 (2.30)	4.25	1.39		28.04	2.96	
**Family type**							
Nuclear	305 (88.9)	4.06	1.15	0.293	26.72	4.91	0.303
Joint	38 (11.1)	4.26	1.08		27.47	4.11	
**Family size**							
≤ 5 members	219 (63.8)	4.03	1.16	0.316	27.09	4.98	0.150
> 5 members	124 (36.2)	4.16	1.11		26.31	4.51	
**Monthly income**							
≤ 5000 BDT	177 (51.6)	3.84	0.94	**0.000**	24.60	4.43	**0.000**
> 5000 BDT	166 (48.4)	4.34	1.29		29.16	4.07	
**Having refrigerator**							
Yes	28 (8.20)	4.57	1.14	**0.017**	31.00	3.06	**0.000**
No	315 (91.8)	4.03	1.14		26.43	4.78	
**Sources of dietary/nutrition information**							
Health professional	84 (24.5)	4.90	1.24	**0.000**	28.48	5.13	**0.000**
Traditional media	10 (2.9)	4.20	0.79		27.20	2.35	
Others^a^	93 (27.1)	4.03	1.06		23.76	4.99	
Don’t know	156 (45.5)	3.65	0.91		27.69	3.79	

*M* Mean; *SD* Standard Deviation

^a^ Others included family members, friends, etc.

### HDD and HFS scores of participants

During the COVID-19 pandemic, the mean HDD score of study participants was 4.08 (SD = 1.15, range: 2 to 7). When HDD scores were analyzed as three categories, 39.1% of households reported low dietary diversity, 56.3% reported moderate dietary diversity, and only 4.7% had high dietary diversity. The mean HFS score of families of day laborers during the COVID-19 pandemic was 26.80 (SD = 4.83, range: 18 to 36). For the food groups consumed, cereals had the highest consumption rate (100%), followed by tuber and roots (73.2%) and vegetables (68.2%), whereas milk and other dairy products (6.4%) were the least consumed (Fig. 1).

### Factors determining the HDD and HFS score

The mean HDD score was significantly higher in participants with a monthly household income of > 5000 BDT (4.34 vs. 3.84, *p* < 0.001) and households with a refrigerator (4.57 vs. 4.03, *p* < 0.05) (Table 1). The adjusted regression model shows that households having a head of household aged > 40 years (Beta coefficient (β) = 0.316, *p* < 0.01), and having monthly income of above 5000 BDT (β = 0.648, *p* < 0.001), were significantly associated with higher score of HDD. Receiving information about dietary/nutrition from health professionals (β = 1.349, *p* < 0.001) and other sources such as family members or friends (β = 0.591, *p* < 0.001) were positively associated with HDD scores, compared to not having any dietary/nutrition awareness (Table 2).

**Table 2:** Associations between socio-demographic variables, and household dietary diversity scores and household food security scores of Bangladeshi day laborers

Variables	HDD score*			HFS score**		
	**β**	**SE**	**p**	**β**	**SE**	**p**
**Residence**						
Rural	Ref	Ref		Ref	Ref	
Urban	-0.076	0.111	0.493	0.133	0.441	0.763
**Age in years**						
≤ 40 years	Ref	Ref		Ref	Ref	
> 40 years	0.316	0.115	**0.007**	0.394	0.461	0.393
**Sex of family head**						
Male	0.167	0.325	0.607	2.184	1.296	0.093
Female	Ref	Ref		Ref	Ref	
**Education of family head**						
No schooling	Ref	Ref		Ref	Ref	
Below secondary	-0.136	0.142	0.118	0.346	0.565	0.540
Above secondary	0.170	0.372	0.648	1.311	0.482	**0.000**
**Family type**						
Nuclear	0.155	0.187	0.408	0.671	0.745	0.369
Joint	Ref	Ref		Ref	Ref	
**Family size**						
≤ 5 members	Ref	Ref		Ref	Ref	
> 5 members	0.226	0.122	0.064	-1.289	0.485	**0.008**
**Monthly income**						
≤ 5000 BDT	Ref	Ref		Ref	Ref	
> 5000 BDT	0.648	0.124	**0.000**	3.826	0.494	**0.000**
**Having refrigerator**						
Yes	0.125	0.206	0.545	1.737	0.822	**0.035**
No	Ref	Ref		Ref	Ref	
**Source of dietary/nutrition information**						
Health professional	1.349	0.138	**0.000**	0.509	0.551	0.357
Traditional media	0.490	0.335	0.145	-1.452	1.337	0.278
Others^a^	0.591	0.138	**0.000**	-0.689	0.551	**0.000**
Don’t get	Ref	Ref		Ref	Ref	

^*^R^2^ = 0.294, Adjusted *R*^2^ = 0.268; ** *R*^2^ = 0.367, Adjusted *R*^2^ = 0.344

*β* Adjusted beta coefficient; *SE* Standard Error

^a^ Others included family members, friends, etc.

The mean HFS score was significantly higher in participants from urban areas than those from rural areas (26.27 vs. 27.36; *p* < 0.05). The mean HFS score was also higher among the participants with a monthly household income of > 5000 BDT (24.60 vs. 29.16, *p* < 0.001) and households with a refrigerator (31.00 vs. 26.43, *p* < 0.001) (Table 1). Results from the adjusted regression analysis indicate that having a household head who completed secondary education (β = 1.311, *p* < 0.001), having monthly income of > 5000 BDT (β = 3.826, *p* < 0.001), and owning a refrigerator (β = 1.737, *p* < 0.005) were positively associated with the HFS scores. On the contrary, households having a family size of > 5 were negatively associated with the HFS scores as compared to those having a family size of ≤ 5 (β = -1.289, *p* < 0.01) (Table 2).

Most of the households’ monthly income was reduced (93.6%), and 97.7% of participants reported increases in the cost of food during the COVID-19 pandemic. The majority of households reported a decrease in both the quantity of food (76%) and variability of food groups (94.8%) they consumed following COVID-19. The mean HDD and HFS scores were significantly lower among the participants who reported that they lost their job and their income was reduced during the COVID-19 pandemic (Table 3).

**Table 3:** Distribution of household dietary diversity and household food security scores of participants across the variables relating impact of COVID-19 (*N* = 343)

Variables	N (%)	HDD score			HFS score		
		**M**	**SD**	**p**	**M**	**SD**	**p**
**Effect of COVID-19 on occupation of HH/earning person of family**							
Same as before	249 (72.6)	4.23	1.19	**0.000**	27.19	4.93	**0.000**
Lost work	18 (5.2)	3.56	0.89		21.33	3.46	
Occupation switched	76 (22.2)	3.71	0.92		26.84	3.93	
**Effect of COVID-19 on income of HH/earning person of family**							
Same as before	22 (6.4)	5.00	1.16	**0.000**	29.64	3.13	**0.004**
Less than before	321 (93.6)	4.02	1.12		26.61	4.86	
More than before	0 (0.0)	-	-		-	-	
**Increase of food prices during the COVID-19 pandemic**							
Yes	335 (97.7)	4.06	1.15	0.094	26.74	4.85	0.110
No	0 (0.0)	-	-		-	-	
Don’t know	8 (2.3)	4.75	0.89		29.50	2.20	
**Get same amount of food as before COVID-19**							
Yes	82 (23.9)	4.95	1.09	**0.000**	30.27	3.65	**0.000**
No	261 (76.1)	3.80	1.02		25.72	4.64	
**Get same type of food as before COVID-19**							
Yes	18 (5.2)	5.00	1.19	**0.000**	31.56	3.60	**0.000**
No	325 (94.8)	4.03	1.12		26.54	4.75	

*M* Mean, *SD* Standard Deviation, *HH* Household Head

Adjusted regression analysis showed that families with households with heads who lost work during the pandemic were negatively associated with HDD score (β = -0.370, *p* < 0.01) and HFS score (β = -4.610, *p* < 0.001), compared to those with stable employment throughout the pandemic. Additionally, households reporting a decrease in the quantity of food resulting from the pandemic were negatively associated with HDD score (β = -1.026, *p* < 0.001) and HFS score (β = -3.955, *p* < 0.001), compared to those whose food consumption remained the same as before the COVID-19 pandemic (Table 4).

**Table 4:** Association between the impacts of COVID-19 pandemic, and the household dietary diversity scores and household food security scores of Bangladeshi day laborers

Variables	HDD score*			HFS score**		
	**β**	**SE**	**p**	**β**	**SE**	**p**
**Effect of COVID-19 on occupation of HH/earning person of family**						
Same as before	Ref	Ref		Ref	Ref	
Lost work	-0.370	0.137	**0.007**	-4.610	1.060	**0.000**
Occupation switched	-0.358	0.254	0.160	0.208	0.571	0.716
**Effect of COVID-19 on income of HH/earning person of family**						
Same as before	Ref	Ref		Ref	Ref	
Less than before	-0.057	0.260	0.828	0.981	1.085	0.367
More than before	-	-		-	-	
**Increase of food prices during the COVID-19 pandemic**						
Yes	-0.310	0.374	0.409	-1.824	1.561	0.243
No	-	-		-	-	
Don’t know	Ref	Ref		Ref	Ref	
**Get same amount of food as before COVID-19**						
Yes	Ref	Ref		Ref	Ref	
No	-1.026	0.149	**0.000**	-3.955	0.622	**0.000**
**Get same type of food as before COVID-19**						
Yes	Ref	Ref		Ref	Ref	
No	-0.191	0.278	0.492	-2.509	1.158	**0.031**

^*^R^2^ = 0.207, Adjusted *R*^2^ = 0.193; ** *R*^2^ = 0.222, Adjusted *R*^2^ = 0.209

*β* Adjusted beta coefficient, *SE* Standard Error, *HH* Household Head

Another linear regression analysis showing the socio-demographic and COVID-19 related factors associated with HDD and HFS scores of day laborers based on residence (urban and rural) were employed so that the policy makers could be given an idea of what to look for in reducing food insecurity and to enhance dietary diversity in urban and rural areas in Bangladesh (see Supplementary Table 1 & 2).

## Discussion

The COVID-19 pandemic has generated a deep crisis for vulnerable populations, including day laborers. In the face of the pandemic, day laborers are a highly vulnerable group for whom job insecurity brings about a financial crisis, which is likely to lower dietary diversity for their households [26]. The current study assessed HDD and HFS associated factors among day laborers, highlighting the dire situation of food insecurity this population faces due to COVID-19.

### Demographic determinants of HFS and HDD

The mean HFS score was significantly higher in urban households than rural households, indicating that the pandemic has compounded food insecurity risk for rural residents. This finding is consistent with a recent report showing a 15% drop in having three meals per day in rural regions during COVID-19 compared to pre-pandemic [27]. The present study found that higher monthly income is a significant predictor of higher HFS, which aligns with a prior Bangladeshi survey indicating that one unit's rise in the householder's farm revenue would raise the possibility of households’ food security [28]. Additionally, households with a monthly income above 5000 BDT had higher HDD status. Households with income levels above this threshold can purchase more varied food from markets [29], while poor households may restrict their food intake to an insufficient number of food products [30]. Furthermore, another Bangladeshi study found about half of the families’ income fell below $1·90 per day, which was a significant risk factor for food insecurity [17].

The current study also found that households with a refrigerator had both higher HFS and HDD scores, which is consistent with past research [11]. Refrigerators provide households the ability to safely preserve a wider variety of food groups for longer durations decreasing the need for more frequent and repeated purchases, which in the face of COVID-19 related challenges allows households more flexibility and opportunistic in food consumption. Our findings align with a recent Bangladeshi study during the COVID-19 pandemic, whereby owning a refrigerator was associated with higher HFS [11]. Owning a refrigerator as a predictive factor of HFS and HDD could be partially explained with refrigerators indicating a component of household income, which, as previously discussed, is associated with increased higher HFS and HDD. Consistent with prior Bangladeshi studies [11], we found that families with a household head with secondary education were more food secure in comparison to those with no formal education. Another study argued that households with less-educated household heads are more likely to be food insecure [31]. Another possible explanation could be that household heads with secondary education might be more aware of their food consumption from variety of food groups as well as their food security. This study is consistent with past literature, indicating that larger family size is associated with increased vulnerability for food insecurity [28], resulting in higher costs of food for more individuals. Respondents who exhibited knowledge about nutrition from health professionals and other sources (such as family members, friends) had greater HDD and HFS scores, compared to those with no basis of dietary/nutrition knowledge, highlights the importance of education and awareness-raising surrounding nutrition as a driver for improved household food security and diversity [12]. Health professionals provide accurate and evidence-based information, whereas other sources of knowledge (e.g., traditional media) often circulate mis-information which explains the higher dietary diversity among respondents having knowledge gained from health professionals.

### COVID-19 related determinants of HFS

We found that decreased household income was a significant risk factor of food insecurity during the COVID-19 pandemic. The economic stressors induced by the impact of various social distancing measures on job and income. Income security is crucially threatening food security among low-income households, including day laborers’ families, who entirely depend on daily income [10, 32, 33]. Financial losses can affect nearly half of the world severely, with extreme poverty and substantially high food insecurity [17]. In the present study, individuals who lost their daily work were more vulnerable to food insecurity during the COVID-19 pandemic compared to those with job security throughout the pandemic. This finding supports a prior Bangladeshi study observed that employment loss results in almost half the population experiencing severe poverty, dramatically rising food insecurity [11, 17]. This situation is affecting day laborers extraordinarily, as it was previously found that 50% of daily wage workers were not permitted to work due to COVID-19 related measures [34].

In the present study, about 76.1% of households reported that they didn’t get the same amount of food and 94.8% reported that they didn’t get the same type of food as before COVID-19. The high rates of impact on food security are consistent with a recent survey which demonstrated that 91% of the total respondents lacked finances for food, while 75% reported not having enough food [26]. Moreover, decreased access to quantity and diversity of food were significantly associated with lower HFS scores, coinciding with a previous study [11]. Unemployment and under-employment, especially in low-income groups like day laborers during the COVID-19 related lockdown and other travel restrictions, decrease the ability to purchase food and disrupt the quality and quantity of food consumed [35].

### COVID-19 related determinants of HDD

The present study showed that losing the daily work of day laborers was a potential risk factor for decreasing HDD status, which is expected as negative economic impacts disrupt food access [36]. In addition, income reduction of the households during the pandemic was also a risk factor for low HDD. Decreasing income may reduce the ability to spend on consumption which might be responsible for lower dietary diversity among participants [30]. Self-employed workers, such as day-to-day workers, do not have access to unemployment benefit schemes or salary pay schemes which leaves them highly vulnerable in terms of supplementing pandemic-related income losses and directly affecting their dietary diversity [10].

Smaller food quantity in comparison to pre-pandemic was also associated with lower HDD scores. Notably, almost all respondents reported increased food prices and reduction in income (97.7% and 93.6%, respectively), which compounds the financial ability to attain HDD with households earning less yet being charged more. The inflation of food markets further increases the vulnerability of low-income households prone to lower dietary diversity [37]. COVID-19 associated measures disturbed the supply chain, likely contributing to global food shortages [38, 39], which can drive the lack of availability of food groups, leading to the decreased dietary diversity among a significant percentage of participants in the study [39]. The ongoing household food insecurity and reduced HDD should be addressed with targeted intervention (i.e., food aid) and continued monitoring of nutritional practices in the food-vulnerable group, including day laborers [40].

### What this study adds

To provide baseline information of the situation, this study has assessed the impact of COVID-19 related socio-economic threats and its impact on HFS and HDD among Bangladeshi day-laborers. Since the information was collected through the face-to-face interview method, the self-reported biasness was limited in the current study. Since there is no existing literature depicting how the pandemic affects low-income groups such as day laborers in Bangladesh. Researchers, nutrition, and health professionals could use the current study results and policymakers to design targeted food security programs informed by a robust evidence base. The present study concluded that the effect of COVID-19 on households facing unemployment has directly impacted socio-economic status and food insecurity, identifying associated factors with food security and dietary diversity. The current study data provide useful information for exploring some of the immediate implications of the COVID-19 crisis, which should be expanded upon through representative and longitudinal samples.

### Limitations

This cross-sectional nature of the study doesn’t allow us to establish causal relationships. Due to dealing with the specific subject group (i.e., day laborers) of this study, the findings may not be generalized into other subject groups (such as rural people, tribal people, employed groups, etc.). Although the HDD scores can help determine food accessibility, it does not capture the amount of actual food consumption by households. Besides, we didn’t measure the pre-pandemic HDD and HFS status; thus, the findings cannot say the causal reason for lower HDD and HFS due to the COVID-19 pandemic.

## Conclusions

The present study identified that a monthly household income of > 5000 BDT ensures both HDD and HFS among the families of day laborers during the pandemic in Bangladesh. Besides, having a family size of > 5 was a potential risk factor of lower HFS score; however, having HH with above secondary education and having a refrigerator were associated with higher HFS score. Similarly, loss of occupation and income reduction were the major risk factors for both low HDD and HFS score during the pandemic situation. Facing current high levels of food insecurity and low dietary diversity during the pandemic, day-laborers in Bangladesh are at risk of long-term nutritional consequences, including micronutrient deficiencies. These nutritional consequences may also lead to significant health-related comorbidities and complications that may intensify their vulnerability to diseases, including COVID-19. It will be necessary that the government and public health agencies recognize this need and respond by introducing effective and sustainable interventions targeting these most vulnerable populations in meeting their basic needs. Thus, this improved understanding of households' food security and dietary diversity status can act as a catalyst for the Government, national and international organizations to reduce the adverse effects of the COVID-19 pandemic on population health and quality of life.

## Supplementary Information

**Additional file 1: Supplementary Table 1. **Associations between socio-demographic variables, and household dietary diversity scores and household food security scores based on residence (rural and urban). **Supplementary Table 2. **Association between the impacts of COVID-19 pandemic, and the household dietary diversity scores and household food security scores based on residence (rural and urban).

## Data Availability

The data and materials underlying this article will be shared on reasonable request to the corresponding author.

## Abbreviations

HDD: Household Dietary Diversity
HFS: Household Food Security
BDT: Bangladeshi Taka
FANTA: Food and Nutrition Technical Assistance
COVID-19: Corona Virus Disease-2019
