# Supplementary material for: Factors associated with household food insecurity and dietary diversity among day laborers amid the COVID-19 pandemic in Bangladesh
Source: BMC Nutr. 2022 Mar 23;8:25. doi: 10.1186/s40795-022-00517-8 (PMC8941835; doi:10.1186/s40795-022-00517-8)
Supplement: Supplementary file 1 — Additional file 1: Supplementary Table 1. Associations between socio-demographic variables, and household dietary diversity scores and household food security scores based on residence (rural and urban). Supplementary Table 2. Association between the impacts of COVID-19 pandemic, and the household dietary diversity scores and household food security scores based on residence (rural and urban). [file 40795_2022_517_MOESM1_ESM.docx]

**Supplementary Table 1** Associations between socio-demographic variables, and household dietary diversity scores and household food security scores based on residence (rural and urban)

| Variables | HDD score | | | | HFS score | | | |
| --- | --- | --- | --- | --- | --- | --- | --- | --- |
|  | **Rural** | | **Urban** | | **Rural** | | **Urban** | |
|  | **β** | **p** | **β** | **p** | **β** | **p** | **β** | **p** |
| **Age in years** |  |  |  |  |  |  |  |  |
| ≤ 40 years | Ref. |  | Ref. |  | Ref. |  | Ref. |  |
| > 40 years | 0.385 | **0.012** | 0.202 | 0.252 | 1.898 | **0.003** | -1.122 | 0.082 |
| **Sex of family head** |  |  |  |  |  |  |  |  |
| Male | 0.656 | 0.238 | 0.056 | 0.893 | 4.376 | 0.062 | 0.728 | 0.631 |
| Female | Ref. |  | Ref. |  | Ref. |  | Ref. |  |
| **Education of family head** |  |  |  |  |  |  |  |  |
| No schooling | Ref. |  | Ref. |  | Ref. |  | Ref. |  |
| Below secondary | -0.378 | 0.055 | -0.355 | 0.090 | 1.382 | 0.095 | -0.674 | 0.375 |
| Above secondary | -0.393 | 0.453 | 0.564 | 0.299 | 3.131 | 0.157 | 9.650 | **0.000** |
| **Family type** |  |  |  |  |  |  |  |  |
| Nuclear | 0.442 | 0.112 | -0.173 | 0.516 | 1.354 | 0.247 | 0.305 | 0.753 |
| Joint | Ref. |  | Ref. |  | Ref. |  | Ref. |  |
| **Family size** |  |  |  |  |  |  |  |  |
| ≤ 5 members | Ref. |  | Ref. |  | Ref. |  | Ref. |  |
| > 5 members | 0.263 | 0.126 | 0.116 | 0.528 | -1.769 | **0.015** | -0.705 | 0.291 |
| **Monthly income** |  |  |  |  |  |  |  |  |
| ≤ 5000 BDT | Ref. |  | Ref. |  | Ref. |  | Ref. |  |
| > 5000 BDT | 0.758 | **0.000** | 0.451 | **0.024** | 3.292 | **0.000** | 4.722 | **0.000** |
| **Having refrigerator** |  |  |  |  |  |  |  |  |
| Yes | 0.899 | **0.018** | -0.192 | 0.459 | 0.203 | 0.898 | 2.233 | **0.019** |
| No | Ref. |  | Ref. |  | Ref. |  | Ref. |  |
| **Source of dietary/nutrition information** |  |  |  |  |  |  |  |  |
| Health professional | 0.612 | 0.109 | 0.392 | 0.602 | -1.620 | 0.312 | 0.392 | 0.886 |
| Traditional media | 1.331 | **0.000** | 1.470 | **0.000** | -0.085 | 0.917 | 0.914 | 0.219 |
| Others^†^ | 0.614 | **0.001** | 0.599 | **0.006** | -3.604 | **0.000** | -1.665 | **0.036** |
| Don’t get | Ref. |  | Ref. |  | Ref. |  | Ref. |  |
| β = Adjusted beta coefficient; SE = Standard Error  ^†^ Others included family members, friends, etc. | | | | | | | | |

**Supplementary Table 2** Association between the impacts of COVID-19 pandemic, and the household dietary diversity scores and household food security scores based on residence (rural and urban)

| Variables | HDD score | | | | HFS score | | | |
| --- | --- | --- | --- | --- | --- | --- | --- | --- |
|  | **Rural** | | **Urban** | | **Rural** | | **Urban** | |
|  | **β** | **p** | **β** | **p** | **β** | **p** | **β** | **p** |
| **Effect of COVID-19 on occupation of HH/earning person of family** | | |  |  |  |  |  |  |
| Same as before | Ref. |  | Ref. |  | Ref. |  | Ref. |  |
| Lost work | -0.546 | **0.004** | -0.135 | 0.507 | -3.380 | **0.011** | -5.470 | **0.001** |
| Occupation switched | -0.430 | 0.205 | -0.261 | 0.498 | 1.335 | 0.068 | -0.706 | 0.416 |
| **Effect of COVID-19 on income of HH/earning person of family** | | |  |  |  |  |  |  |
| Same as before | Ref. |  | Ref. |  | Ref. |  | Ref. |  |
| Less than before | 0.001 | 0.998 | -0.305 | 0.581 | 0.164 | 0.887 | 1.648 | 0.482 |
| More than before | - | - | - | - | - | - | - | - |
| **Increase of food prices during the COVID-19 pandemic** | |  |  |  |  |  |  |  |
| Yes | -0.530 | 0.383 | -0.181 | 0.712 | 1.338 | 0.573 | -3.085 | 0.139 |
| No | - | - | - | - | - | - | - | - |
| Don’t know | Ref. |  | Ref. |  | Ref. |  | Ref. |  |
| **Get same amount of food as before COVID-19** | |  |  |  |  |  |  |  |
| Yes | Ref. |  | Ref. |  | Ref. |  | Ref. |  |
| No | -0.873 | **0.000** | -1.360 | **0.000** | -4.479 | **0.000** | -4.377 | **0.000** |
| **Get same type of food as before COVID-19** | |  |  |  |  |  |  |  |
| Yes | Ref. |  | Ref. |  | Ref. |  | Ref. |  |
| No | -0.261 | 0.416 | -0.032 | 0.953 | -2.346 | 0.063 | -2.713 | 0.240 |
| β = Adjusted beta coefficient; SE = Standard Error; HH = Household Head | | | | | |  |  |  |
